# The scientific method: pillar and pitfall of cancer research

**DOI:** 10.1002/cam4.248

**Published:** 2014-04-08

**Authors:** Shi-Ming Tu, Mehmet Asim Bilen, Nizar M Tannir

**Affiliations:** Department of Genitourinary Medical Oncology, Unit 1374, The University of Texas MD Anderson Cancer CenterP.O. Box 301439, 1155 Pressler Street, Houston, 77230-1439, Texas

A major goal of cancer research is to acquire knowledge that enables us to understand cancer and help mankind. To reach this goal, we need to conduct cancer research according to the scientific method. The scientific method stipulates that we begin by making observations of cancer. Then, we formulate hypotheses to explain our observations and devise experiments to test our hypotheses. Importantly, we need to validate the results of these experiments by performing independent verifications. However, many of us have strayed from the scientific method in our research. We often focus on observations from the laboratory rather than from the clinic. Unfortunately, the hypotheses and experiments derived from such observations may be impertinent, and the results could be misconstrued and self-serving. We propose that cancer research is more likely to reach its goal of understanding cancer and helping mankind, if it adheres to the basic principles of the scientific method.

## Cancer Research

Begley and Ellis reminded us that only 11% of our “landmark” research studies are reproducible 1. They attributed our failures to inadequate controls, selective data presentation, and lack of appropriate cell lines or animal models, and advocated ways to remedy this shortcoming. We assert that the root of our current “low” standard of preclinical research is much more fundamental. To rectify this glaring scientific misadventure, we need to resurrect the scientific method.

The scientific method requires us to formulate theories to elucidate our myriad intriguing observations of cancer. A useful theory empowers us to ask the right questions and pose pertinent hypotheses. It ensures that we design proper experiments and select appropriate models, and enables us to make sound interpretation of laboratory results. Most importantly, a useful theory will expedite the discovery of effective therapies, which will be the ultimate proof of its veracity.

## The Scientific Method: A Brief History

Aristotle (384–322 B.C.E.), the great Greek philosopher and teacher, laid the foundation of modern science by establishing an objective method for acquiring knowledge through reason. In particular, he formulated the basic principles of scientific epistemology: the role of the senses, the role of abstraction, the laws of logic, the types of reasoning, and the basic rules of validity in deductive reasoning.

Robert Grosseteste (1175–1253 C.E.), an English scientist, statesman, and bishop, elaborated on Aristotle's ideas and laid the basic framework for the scientific method: generalizing from particular observations to a universal law, and then using the universal law to predict particular observations. He believed that both efforts must be verified by experimentation.

Roger Bacon (1214–1294 C.E.), an English monk, was inspired by the writings of Grosseteste and others. During his time, many people downplayed and even condemned scientific explanations of the world. Traditional authorities dominated people's views and understanding of the world. Bacon prescribed a scientific method that is based on a repeating cycle of observation, hypothesis, experimentation, and verification. For his efforts, some people considered him to be the father of modern science.

## Cancer Controversies

When we fail to adhere to the basic principles of the scientific method, cancer research is prone to misdirection. Such research produces conflicting results that instigate controversy. Ironically, a controversy is good for science in its own ways. It indicates that we have an important problem that requires insightful solutions.

### Cancer stem cells

The existence of cancer stem cells is controversial. Bonnet and Dick performed a classic experiment that demonstrated the presence of cancer stem cells using an in vivo clonal assay 2, but Quintana et al. performed an alternative experiment that questioned the existence of these very same cells using a similar assay in a different animal model 3.

A key observation of cancer pertinent to the controversy of cancer stem cells is that people who develop cancer almost always have intact immune systems 4–6. Using an immune-compromised animal to test the hypothesis of cancer stem cells (as Bonnet and Dick did)^2^ seems acceptable, because that is what experiments are designed to do—test a specific hypothesis. But using an even more immune-compromised animal (as Quintana and colleagues did)^3^ to refute the results of or a hypothesis generated from another experiment—rather than test an alternative hypothesis— is inherently misguided.

If similar experiments were performed in immune-competent animals, then the results would have been more revealing. Indeed, Zhao et al. 7. demonstrated that tumors derived from induced pluripotent stem cells were duly recognized and rejected by the immune system of immune-competent syngeneic mice, whereas those that originated from embryonic stem cells formed teratomas. It is evident that this is a more relevant experimental model to investigate what actually happens to naturally occurring cancers in humans.

### Dedifferentiation

There is also controversy regarding dedifferentiation of cancer. Does cancer dedifferentiate from differentiated somatic cells, or does a poorly differentiated cancer merely reveal its undifferentiated (i.e., “stemness”) phenotype? Goldstein et al. demonstrated that differentiated acinar cell is not the origin of prostate cancer 8, but Schwitalla et al. showed that dedifferentiation and acquisition of stem cell-like properties initiate intestinal tumorigenesis 9.

A pivotal observation of cancer relating to the controversy of dedifferentiation is that most somatic differentiated cells have a short life span. There is simply insufficient time for multiple mutations to occur and accumulate in a somatic cell with a limited life span (e.g., skin, 30 days; gut, 3 days). Perhaps certain somatic cells contain stemness properties, but this argument defies the very essence of dedifferentiation. When we accept the role of experimentation in the scientific method, we minimize the chance that results from experiments become self-fulfilling or are artifacts.

Importantly, hypotheses dealing with a human condition need to be tested and verified in human samples or cases. Hence, Penney et al. demonstrated that the Gleason grade does not progress over time suggesting that human prostate cancer does not de-differentiate 10. Som et al. showed that it is more likely for undifferentiated spermatogonial stem cell-like cells to form a seminoma than differentiating spermatogonia or differentiated spermatozoa (with a life span of less than 3 months) to dedifferentiate and form a seminoma in humans 11,12. After all, prostate cancer and testicular seminoma are unique human cancers.

## Paradigm Shift

Unfortunately, when we do not adhere to the basic principles of the scientific method, we lose perspective about the role of experiments. We live in a culture that embraces the mentality that all experiments are equal and all results are factual. We believe that all experiments have merit and that all the results derived from them provide useful information, even though the main purpose of all experiments is to test their respective hypotheses, which may or may not pertain to any pertinent observations of nature. In short, we all seem to accept too literally and readily the results of these experiments without fully understanding their true worth or real meaning in the greater context of the scientific method in cancer research.

A major paradigm shift is in order regarding translational research. Indeed, the conventional wisdom that clinical research is best done from the bench to the bedside may be ill-advised 4. When we conduct clinical research in this manner, we tend to focus on and emphasize the design and execution of experiments and overlook what observations of nature we are actually addressing and what hypotheses of pertinence we are testing. Instead, we propose that clinical research will be more rewarding and informative when it is performed from the bedside (where we make seminal observations and conceive relevant hypotheses) to the bench and then back to the bedside. We contend that conducting translational research without the guidance of a scientific method is a grave mistake.

## Conclusion

It is unfortunate that many scientists are conducting preclinical cancer research without strict adherence to the scientific method. It is even more unfortunate that many patients are receiving treatments with marginal benefit-to-risk ratios based on questionable translational research as a consequence of this practice. We need to formulate plausible hypotheses based on pertinent observations derived from the clinic rather than from the laboratory. After all, experiments are designed to test, not to generate, hypotheses. By adhering to the scientific method, we will create a higher standard of preclinical research and produce more actionable scientific facts rather than misleading scientific fallacies.
